# Treatment of Extraneural Metastases of Myxopapillary Ependymomas With Dose-Dense Temozolomide and Lapatinib

**DOI:** 10.7759/cureus.67928

**Published:** 2024-08-27

**Authors:** Devon C Riegel, Ekokobe Fonkem, Jennifer M Connelly

**Affiliations:** 1 Neurology, Medical College of Wisconsin, Milwaukee, USA

**Keywords:** lapatinib, pulmonary metastases, sacrococcygeal ependymoma, extraneural metastases, recurrent ependymoma, myxopapillary ependymoma

## Abstract

Myxopapillary ependymomas (MPEs) are rare tumors of the central nervous system, and outcomes are generally worse with recurrent disease. These tumors can rarely metastasize outside the neuraxis. We present a case of a 35-year-old female with a history of MPEs who developed extraneural metastases 11 years after her initial gross total resection. Sites of metastases included multiple bilateral intrapulmonary and pleural-based masses with pleural effusion and a pelvic mass. The patient was treated with dose-dense TMZ and lapatinib and had a mixed radiographic response after 12 cycles of treatment. This is the first known case of extraneural metastases of MPEs to demonstrate a radiographic response to dose-dense TMZ and lapatinib. This case presentation discusses the need to establish optimal treatment of extraneural ependymal metastases, duration of treatment, and strategy for the management of recurrent diseases.

## Introduction

Myxopapillary ependymomas (MPEs) are recently reclassified World Health Organization (WHO) grade 2 tumors that usually affect the sacral spinal cord [1]. MPEs have a reported annual incidence of approximately 0.05-0.08 in 100,000 people [2,3]. MPEs have a high recurrence rate, especially if gross total resection (GTR) is not achieved [4,5]. Recurrence tends to be local but distant central nervous system (CNS) metastases are not uncommon. Rarely, MPEs can metastasize to extraneural locations, including pelvic structures (i.e., uterus, ovaries) and the lungs [4,6-12]. The sacro-coccygeal region is the most common origin of ependymomas that develop extraneural metastases, and of those, 46.5% are MPEs [11].

There is an absence of consensus on the best treatment approach for extraneural MPE metastases [4,7]. The standard of care for initial MPE diagnosis is GTR, or subtotal resection with radiotherapy (RT) [3,4,7,13]. Systemic treatment for CNS dissemination or extraneural metastasis has shown inconclusive results regarding improvement of clinical outcomes [8,11-14]. A recent phase II clinical trial demonstrated the efficacy of using dose-dense temozolomide (TMZ) and lapatinib for the treatment of metastatic, recurrent ependymomas. Lapatinib was selected due to frequent ErB1 (EGFR) and ErB2 (HER2) mutations in ependymal tumors [14]. However, none of the patients involved in that study presented with extraneural ependymoma recurrence.

We present a case of a 35-year-old female with a history of MPEs who developed extraneural metastases 11 years after her initial GTR and was treated with dose-dense TMZ and lapatinib with a mixed radiographic response throughout 12 cycles of treatment. This case was previously presented as a meeting abstract at the 2022 Society of Neuro-Oncology Annual Scientific Meeting on November 14, 2022 [15].

## Case presentation

**Figure 1:**
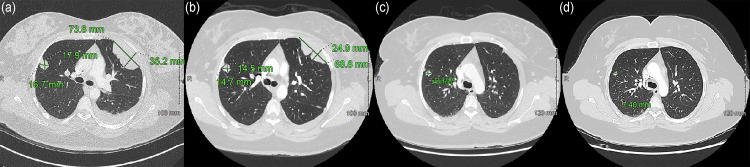
Chest CT demonstrating (a) two initial pulmonary masses, (b) post-cycle 3, (c) post-cycle 6, and (d) post-cycle 12 changes to pulmonary masses.

**Figure 2:**
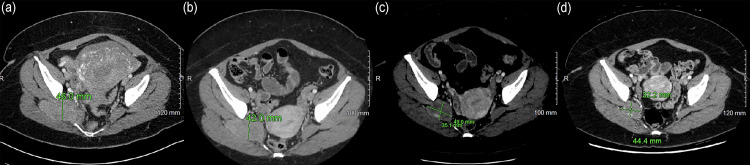
Abdominal and pelvic CT demonstrating (a) initial pelvic mass affecting the right sciatic nerve, (b) post-cycle 3, (c) post-cycle 6, and (d) post-cycle 12 changes to the pelvic mass.

A 35-year-old female presented with shortness of breath and a history of MPE treated with GTR 11 years prior. A chest CT revealed multiple bilateral intrapulmonary and pleural-based masses with pleural effusion (Figure 1a). The patient also described pain that radiated from her right gluteal region to her right knee during activity. Abdominal and pelvic CT revealed another mass involving the right sciatic nerve in the gluteal region, likely secondary to perineural spread from her original tumor site (Figure 2a). An MRI of the brain and spine were negative for disease, and her original site of disease in the sacro-coccygeal region showed no evidence of recurrence.

Biopsies were performed on the pelvic mass and one of the intrapulmonary nodules. Histology of the pelvic mass demonstrated round cells with elongated nuclei and eosinophilic cytoplasm arranged in perivascular pseudorosettes in the myxoid matrix, which was consistent with MPE. Methylation profiling was positive and also consistent with MPE. Immunohistochemistry (IHC) of the pelvic mass stained positive for glial fibrillary acidic protein (GFAP), EGFR, Nectin-4, and weakly positive Trop-2, while negative for ER, AR, HER2, FOLR1, Claudin 18.2, PD1 and PD-L1. GlioSeq testing demonstrated KRAS mutation, copy number gain at 3p, 9p/9q, 13q, 17p/17q, and 19p/19q and copy-neutral loss of heterozygosity at 1p, 6p, 6q, 10a, 16p and 16q. Histology of the pulmonary mass was comparable to that of the pelvic mass and was also diagnosed as MPE. Methylation profiling was attempted but was unable to be completed due to insufficient tissue. IHC demonstrated strongly positive GFAP, weakly positive EGFR (30% of tumor cells), and patchy positivity of synaptophysin. The following IHC stains were negative: EMA, TTF1, PR, napsin, CK5/6, CK8/18, and S100. Next-generation sequencing was performed including Tempus reporting, which demonstrated overexpression of HER2 and Wilms’ tumor 1 (WT1) without additional germline mutations. 

Systemic treatment was delayed due to the patient being 29 weeks pregnant at the time of diagnosis. The patient delivered her baby at 36 weeks gestation, after which systemic treatment was initiated. The patient started oral TMZ 100 mg/m^2^ (seven days on, seven days off) and daily lapatinib 1250 mg four weeks postpartum. The patient received a total of 12 cycles over 12 months on a 28-day schedule. In cycle 2, the TMZ dose was escalated to 125 mg/m^2^ but treatment was held after the first week due to pruritic rash. In cycle 3, TMZ and lapatinib doses were reduced to 100 mg/m^2^ and 1000 mg, respectively, due to continued rash. This dosing was maintained for the remaining cycles. Side effects of treatment included acneiform rash and diarrhea. The rash was initially treated with topical clindamycin (after cycle 8) and hydrocortisone, which initially managed symptoms, but effectiveness declined with prolonged use. In the final cycle, the patient’s side effects to treatment progressed to include nausea, decreased appetite, and dizziness, at which point treatment was suspended in favor of ongoing observation at four-month intervals.

Imaging by CT was performed at three-month intervals following treatment induction. The pelvic mass and all pulmonary masses showed an interval decrease in size across consecutive scans (Figures 1, 2). The patient reported a decrease in shortness of breath and right leg pain after cycle 3. After cycle 3, the only persistent symptom experienced by the patient related to disease burden was intermittent right leg neuropathy described as “shock” sensations radiating down the right lower limb from the right hip. This symptom was exacerbated by cold temperatures and increased physical activity but overall improved steadily throughout the course of treatment.

**Figure 3:**
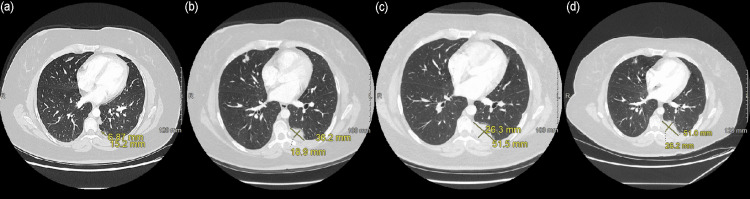
Chest CT demonstrating (a) left lower lobe pleural nodule post-cycle 12, (b) 4 months post-treatment, (c) post-cycle 3 resumption of TMZ/lapatinib and (d) post-radiation treatment changes to the pleural nodule.

Four months after cessation of treatment, follow-up imaging revealed mixed treatment response with stability or decrease in size of the pelvic mass and most pleural nodules, but one left lower lobe pleural nodule increased in size (Figures 3a, 3b). Upon tumor board discussion, TMZ/lapatinib was resumed. Three months after resumption of systemic therapy, chest CT demonstrated continued progression of pleural disease (Figure 3c). The patient also developed progressive respiratory symptoms. Therefore, the patient was treated with 2500 cGy RT in five fractions. Chest CT following RT demonstrated stabilization of pleural disease (Figure 3d). Palliative RT led to four-month durability of disease before the patient experienced interval growth of a pleural nodule. She subsequently trialed trastuzumab and enfortumab with daily lapatinib 750 mg and remains on this treatment regimen at the time of publication.

## Discussion

An MPE is a rare tumor with a low rate of extraneural recurrence. While dose-dense TMZ and lapatinib has shown efficacy for treating recurrent ependymoma within the neuro-axis, there have been no reported cases of this regimen successfully treating extraneural MPE metastases [14]. While some extraneural MPE metastases have shown temporary response to TMZ, there remains a need for a more efficacious regimen to treat disease recurrence [11]. In a case series published by Katz et al. in 2018, a patient with a recurrent extraneural MPE was treated with lapatinib and TMZ but showed disease recurrence after only two cycles [12]. To our knowledge, this is the first known case of extraneural metastases of MPEs to demonstrate response to dose-dense TMZ and lapatinib through 12 cycles. Our patient’s prolonged response to TMZ/lapatinib may be explained by differences in the molecular profile. However, this comparison is limited due to the absence of reported EGFR and HER2 expression in the Katz case. Furthermore, our patient’s original pathology performed 11 years before recurrence was performed outside of the United States and is not available for reinterpretation.

Throughout the initial 12 cycles of systemic therapy, the patient experienced progressive improvement of clinical symptoms as well as disease burden with interval reduction of the pelvic mass and pleural masses. Cessation of treatment led to the progression of pleural disease and rechallenge with systemic therapy alone was not successful in treating disease progression. Tumor recurrence and failure of repeat systemic therapy may be explained by tumor heterogeneity and developed resistance to chemotherapeutic agents [16]. Previous studies have reported mixed results on the efficacy of salvage chemotherapy for the treatment of recurrent ependymoma. Platinum-based chemotherapies have been used in previous reports but demonstrate minimal effects on progression-free survival (PFS) [17-19]. Green et al. (2009) evaluated bevacizumab in a small retrospective cohort in which six of eight patients achieved partial response [20]. Rico et al. (2020) presented a case of a patient with extraneural MPE treated with tislelizumab, an anti-PD1 checkpoint inhibitor [17]. Their patient experienced disease stabilization for 12 months on tislelizumab treatment, receiving a total of 18 months of treatment before trialing oral TMZ [17]. Notably, this patient lacked EGFR mutations, and HER2 mutations were not reported. This case suggests immunotherapy as a potential alternative after TMZ/lapatinib failure or in the absence of EGFR and HER2 overexpression. In the presence of HER2 overexpression, other HER2-targeting agents such as trastuzumab could also be considered.

RT was not initiated at the time of the first recurrence as the patient had a low symptom burden and had a significant response to TMZ/Lapatinib. However, after further progression and development of symptoms, RT was recommended after failure of systemic therapy alone and was successful at achieving stability of her disease. Previous studies suggest that palliative RT can help achieve prolonged PFS in recurrent ependymomas [18,21]. A case published by Cheung et al. (2020) presented a patient with recurrent extraneural ependymoma treated with 28 Gy in 7 daily fractions who showed durability of response for three years before recurring and receiving etoposide systemic therapy [22]. Our patient's case demonstrates the variable efficacy of palliative RT, which stabilized her disease for only a few months. With this case, as with the aforementioned cases of recurrent extraneural ependymoma, many patients will trial several salvage therapies, all of which cannot achieve full remission of disease. There remains a significant need to investigate treatment modalities for recurrent extraneural ependymoma to achieve long-term durability of treatment response.

## Conclusions

In summary, this is a unique case of a patient with a remote history of MPEs treated initially with GTR that recurred outside the neuraxis and showed a positive response to systemic treatment with dose-dense TMZ and lapatinib. This case highlights a need to explore optimal treatment of extraneural ependymal metastases, duration of treatment, and management of recurrent disease. Because of the low incidence of extraneural MPE, randomized clinical trials are generally not feasible. Potential future studies could assess dose-dense TMZ and lapatinib in a small cohort of extraneural ependymal tumor patients.

## References

[1] Louis DN, Perry A, Wesseling P (2021). The 2021 WHO Classification of Tumors of the Central Nervous System: a summary. Neuro Oncol.

[2] Weber DC, Wang Y, Miller R (2015). Long-term outcome of patients with spinal myxopapillary ependymoma: treatment results from the MD Anderson Cancer Center and institutions from the Rare Cancer Network. Neuro Oncol.

[3] Scarpelli DB, Turina CB, Kelly PD, Khudanyan A, Jaboin JJ, McClelland S 3rd (2020). National trends in management of adult myxopapillary ependymomas. J Clin Neurosci.

[4] Fujimori T, Iwasaki M, Nagamoto Y, Kashii M, Sakaura H, Yoshikawa H (2013). Extraneural metastasis of ependymoma in the cauda equina. Global Spine J.

[5] Nakamura M, Ishii K, Watanabe K, Tsuji T, Matsumoto M, Toyama Y, Chiba K (2009). Long-term surgical outcomes for myxopapillary ependymomas of the cauda equina. Spine (Phila Pa 1976).

[6] Batich KA, Riedel RF, Kirkpatrick JP (2019). Recurrent extradural myxopapillary ependymoma with oligometastatic spread. Front Oncol.

[7] Seo SH, Paul SK, Shikder M (2021). An insight into pathophysiological features and therapeutic advances on ependymoma. Cancers (Basel).

[8] Ilhan A, Furtner J, Birner P, Rössler K, Marosi C, Preusser M (2011). Myxopapillary ependymoma with pleuropulmonary metastases and high plasma glial fibrillary acidic protein levels. J Clin Oncol.

[9] Güzin K, Bozdağ H, Aydın A, Şahin S, Özkanlı Ş (2016). Uterine cervix metastasis of myxopapillary ependymoma originated from the spinal cord. Balkan Med J.

[10] Whittemore DE, Grondahl RE, Wong K (2005). Primary extraneural myxopapillary ependymoma of the broad ligament. Arch Pathol Lab Med.

[11] Bruno F, Pellerino A, Bertero L, Soffietti R, Rudà R (2020). Long-term survival of a sacro-coccygeal myxopapillary ependymoma with extra-neural metastases: case report and review of the literature. Neurol Sci.

[12] Katz SY, Cachia D, Kamiya-Matsuoka C (2018). Ependymomas arising outside of the central nervous system: a case series and literature review. J Clin Neurosci.

[13] Gerstner ER, Pajtler KW (2018). Ependymoma. Semin Neurol.

[14] Gilbert MR, Yuan Y, Wu J (2021). A phase II study of dose-dense temozolomide and lapatinib for recurrent low-grade and anaplastic supratentorial, infratentorial, and spinal cord ependymoma. Neuro Oncol.

[15] Riegel DC, Connelly JM (2022). INNV-17. Extraneural metastases of myxopapillary ependymoma treated with dose-dense temozolomide and lapatinib. Neuro Oncol.

[16] Zhang A, Miao K, Sun H, Deng CX (2022). Tumor heterogeneity reshapes the tumor microenvironment to influence drug resistance. Int J Biol Sci.

[17] Rico GT, Townsend A, Price T, Patterson K (2020). Metastatic myxopapillary ependymoma treated with immunotherapy achieving durable response. BMJ Case Rep.

[18] Iqbal MS, Lewis J (2013). An overview of the management of adult ependymomas with emphasis on relapsed disease. Clin Oncol (R Coll Radiol).

[19] Brandes AA, Cavallo G, Reni M (2005). A multicenter retrospective study of chemotherapy for recurrent intracranial ependymal tumors in adults by the Gruppo Italiano Cooperativo di Neuro-Oncologia. Cancer.

[20] Green RM, Cloughesy TF, Stupp R, DeAngelis LM, Woyshner EA, Ney DE, Lassman AB (2009). Bevacizumab for recurrent ependymoma. Neurology.

[21] Bouffet E, Hawkins CE, Ballourah W (2012). Survival benefit for pediatric patients with recurrent ependymoma treated with reirradiation. Int J Radiat Oncol Biol Phys.

[22] Cheung BM, Lau JK, Lo AW, Luk MY, Yuen KK (2020). A rare case of metastatic primary peritoneal ependymoma: a case report and literature review. Case Rep Oncol Med.

